# Acute Health Effects and Outcome Following Sarin Gas Attacks in Khan Shaykhun, Syria

**DOI:** 10.7759/cureus.22188

**Published:** 2022-02-14

**Authors:** Osama I Alsaleh, Abdallah M Elsafti Elsaeidy, Saad Saeed, Abdulrahman Alhallak, Mazen A Altelawi, Gerlant Van Berlaer, Ives Hubloue

**Affiliations:** 1 Disaster Medicine, Al-Sham Humanitarian Foundation, Istanbul, TUR; 2 Emergency and Disaster Medicine, Vrije Universiteit Brussel, Brussels, BEL; 3 Emergency, Hamad Medical Corporation, Doha, QAT; 4 Pediatrics, Hamad Medical Corporation, Doha, QAT; 5 Medical Surgical Nursing, Alexandria University, Alexandria, EGY; 6 Nursing, Al-Baath University, Hama, SYR; 7 Pharmacy, Zaparozhye State Medical Univeristy, Zaparozhye, UKR; 8 Emergency and Disaster Medicine, Universitair Ziekenhuis Brussel, Brussels, BEL; 9 Research Group on Emergency and Disaster Medicine, Universitair Ziekenhuis Brussel - Vrije Universiteit Brussel, Brussels, BEL

**Keywords:** public health, khan shaykun attacks, chemical attacks, chemical weapons, sarin gas, syria

## Abstract

Background

In 2017, Idlib, Syria, was exposed to a chemical attack with sarin gas. Many patients of the attack were presented to the Al Rahman Charity Hospital in northern Syria. The aim of this study is to describe the clinical manifestations of sarin gas exposure, as well as the management and outcome of these manifestations in areas with poor healthcare infrastructure.

Methods

In a case series study design, medical records of suspected sarin exposed patients were reviewed in terms of age, gender, initial clinical presentation, management, and outcome.

Results

Seventeen patients with signs of sarin gas exposure had detailed medical records. The mean age was 29.1 years with a range of 4-70 years. Six patients were male (35.3%), and four (23.5%) were children under 18 years.

At initial presentation, all victims suffered from respiratory distress because of severe airway inflammation, chest pain, and ophthalmological symptoms. All patients featured varying degrees of intestinal, neurologic, and dermatological signs and symptoms. Acute symptom management consisted of oxygen (100% of patients), atropine (100%), bronchodilators (82.4%), dexamethasone (82.4%), anti-emetics (82.4%), paracetamol (47.1%), and ranitidine (41.2%).

Rapid symptomatic recovery was observed in 13 patients (76.5%) who stayed in the hospital for less than 24 hours, but four patients (23.5%) had to be admitted for more than 24 hours. The median length of stay was 22.2 hours (with a range of eight to 48 hours). Two patients required intensive care. Of the studied sample, all patients survived.

Interpretation

This study demonstrates that even in austere healthcare settings, survival rate and prognosis of sarin gas contaminated patients are fair if basic measures and symptomatic treatment are performed. The study provides insight into the clinical presentation, management, and hospital course likely to result from future sarin gas releases.

## Introduction

Khan Shaykhun is a town in the Idlib Governorate of Syria that was exposed to a chemical attack on April 4, 2017 [1]. Aircraft dropped munitions over Khan Shaykhun between 6:30 and 7:00 AM, creating a crater from which the sarin gas emanated. A large number of people were affected by the sarin within this timeframe. Victims’ symptoms and their subsequent medical treatments, as well as the scale of the incident, are consistent with large-scale sarin intoxication. The Sarin that was founded in the samples taken from Khan Shaykhun has most likely been made with a precursor (DF) from the original stockpile of the Syrian Arab Republic [1]. It was reported that structured armed forces attacked the town by an airstrike followed by massive civilian chemical poisoning [2,3]. According to the opposition Idlib health authority the released toxic gas, which included sarin or a similar substance [4], killed at least 74 people and injured more than 557 casualties [5,6]. During the Syrian civil war, this attack was the deadliest use of chemical weapons since the Ghouta chemical attack in 2013 [7].

The Organisation for the Prohibition of Chemical Weapons (OPCW) Fact-Finding Mission in the Syrian Arab Republic found that many people had been exposed to sarin or a sarin-like substance during the attack at Khan Shaykhun, based on its analysis of biomedical specimens, interviews, and supplementary material submitted during the interview process, as well as analysis of environmental samples [1].

The Syrian regime has been accused of responsibility for the attack, according to the OPCW-UN Joint Investigative Mechanism (OPCW-UN JIM), which has also described marker chemicals that linked the sarin used to the Syrian government: “The samples from Khan Shaykhun contain the three types of marker chemicals: PF6 (HFP), isopropyl phosphates and isopropyl phosphorofluoridates, their presence is a strong indicator that the sarin disseminated in Khan Shaykhun was produced from methyl phosphonic difluoride (DF) from the Syrian Arab Republic stockpile” [1,8].

The UN Chemical Weapons Convention which is signed by 192 countries has condemned any use of toxic chemicals as a weapon by anyone anywhere in any circumstances. They consider its use as a violation of international law and have expressed their conviction that those responsible should be held accountable. (OPCW fact-finding mission confirms the use of chemical weapons in Khan Shaykhun on April 4, 2017; June 30, 2017 [1,8].)

Khan Shaykhun chemical attack patients were treated at many neighboring hospitals such as Alnoor Hospital in Telmins, Central Ma’arat Alnuman Hospital, Idlib’s National Hospital, and Bab Al-Hawa Hospital. The nearest hospital to the site of the attack was al Rahman Charity Hospital in Al-Tah (15 km from Khan Shaykhun). The hospital has 26 beds, and the hospital staff consists of three doctors and 11 nurses as follows: one trauma surgeon; one pediatrician; one gynecologist; two pharmacy technicians; one laboratory technician; four nurses; one anesthesia technician; one operations technician; and two midwives.

The medical staff at al Rahman has extensive experience in wars and disasters in addition to having undergone special training in the management of chemical weapons exposure through the Chemical, Biological, Radiological, Nuclear-Taskforce (CBRN-Taskforce) [9]. Not all the necessary drugs were available in the hospital. Important medicines such as pralidoxime were not available.

An organophosphate “nerve agent,” sarin was used in two terrorist attacks in Japan in the 1990s, and the effects of the gas on humans were well documented in these two incidents. During the 1994 Matsumoto attack, Sarin gas inhalation caused instantaneous death by the respiratory arrest in several victims, while resuscitation efforts were successful in treating other severely injured victims who were presenting in a coma and generalized convulsions, with rapid recovery and no sequelae. Affected victims have presented commonly with miosis and blurred-dark vision, ocular pain, copious secretions from respiratory and gastrointestinal tracts (muscarinic effects), and headaches. The severity of signs and symptoms in the victims was paralleled to deficiency of plasma cholinesterase (ChE) activity. Treatments including oximes, atropine sulfate, diazepam, and intravenous fluids infusion were shown to be effective in these cases [10].

Sarin (GB, O-isopropyl methyl phosphonofluoridate) is a potent organophosphorus (OP) nerve agent that inhibits acetylcholinesterase (AChE) irreversibly. Accumulation of acetylcholine (ACh) in the central nervous system (CNS) provokes seizures and, at sufficient doses, centrally mediated respiratory arrest [11-13]. Victims exposed to these poisonous agents develop miosis, tightening of the chest, difficulty breathing, and a general loss of bodily function due to the continuous stimulation of muscles, glands, and the CNS. With symptoms progression, victims suffer from convulsive spasms and seizures that can quickly progress to status epilepticus (SE), which is strongly associated with brain damage in survivors, and death [12-15].

Besides its primary action on the cholinergic system, sarin possesses other indirect effects. These involve the activation of several neurotransmitters including gamma-amino-butyric acid (GABA) and the alteration of other signaling systems such as ion channels, cell adhesion molecules, and inflammatory regulators. Exposure to Sarin is associated with symptoms of organophosphate-induced delayed neurotoxicity (OPIDN) and organophosphate-induced chronic neurotoxicity (OPICN). Furthermore, sarin has been shown to create toxic and immunotoxic effects as well as organophosphate-induced endocrine disruption (OPIED) [11].

Therapy after exposure to sarin gas is divided into three categories: decontamination, respiratory support, and antidotes. All of these therapies may be administered simultaneously [16]. Decontamination of the victim involves the use of bleach, soap, and water. Staff handling casualties require protection in the form of respirators and butyl rubber boots and gloves [11,17]. Treatment is based on the use of large doses of atropine, a muscarinic receptor antagonist, accompanied by an oxime, an AChE reactivator, and diazepam, which may lead to practical problems of sufficient drug supplies for the average hospital. Ventilation may be necessary.

Recommended medical countermeasures against toxic levels of nerve agents can increase survival if administered within a short period of time following exposure, but they may not fully prevent neuropathology or functional impairment [13,15,18].

The aim of this study is to describe the clinical manifestations, effects management, and outcomes of sarin gas exposure in areas with poor healthcare infrastructure based on the victims of the 2017 Khan Shaykhun attack. The limitations of this study were also discussed.

## Materials and methods

In a case series study design, medical records of suspected sarin exposed patients were reviewed in terms of age, gender, initial clinical presentation, management, and outcome. The local health directorate in Idlib approved this study.

Khan Shaykhun is both a town and a sub-district of the Maarrat al-Nu'man District within the governorate of Idlib in the northwestern Syrian Arab Republic (35.44° N, 36.65° E, at 376 m above sea level). Located about 10 km away from the border of the Hama governorate to the south and about 100 km away from the Aleppo Governorate to the north, recently available information estimates that the sub-district of Khan Shaykhun has a population of approximately 34,000 people, with the town itself having a population of 16,000 people [1].

On April 4, 2017, during this Sarin gas attack, 17 patients met the criteria of possible sarin gas exposure from those victims who were admitted to al Rahman Charity Hospital. Diagnoses of sarin gas exposure were based on patient complaints and physical examinations performed by a well-trained physician. Patients were eligible for inclusion in the case series if their record was complete regarding initial presentation, evolution, management, and documentation until discharge. It has been confirmed that the Patients do not have allergies or other relevant diseases.

Oral informed consent for participation in this study was obtained from each patient or from a legal guardian in the case of victims under 18 years. By reviewing paper medical records, the authors registered the following for all patients within the study: age, gender, demographics, detailed medical history including previous pathological conditions, allergies, use of routine daily medication, initial and evolving clinical presentation, vital signs (pulse rate, blood pressure, temperature, and respiratory rate), physical features from systematic clinical examination, initiated and sustained management, and outcome. All data were anonymized according to the Helsinki Declaration. Patients with missing or unreadable data were excluded. Descriptive statistics for discrete outcome variables were presented as frequencies and proportions (n; %), and for quantitative variables (age, number of children) as measures of central tendency and dispersion (median, range, interquartile range IQR). The analyses were broken down for age (<18 and ≥18 years old). Analyses were carried out by using IBM® SPSS® v23.0 (IBM Corp., Armonk, NY, USA). All tests were performed using an α-level of 0.05.

## Results

Seventeen patients were admitted to al Rahman Charity Hospital in Al-Tah in the Idlib Governorate of Syria following exposure to toxic gas. All patients presented on the day of exposure, and with similar symptoms. Except for these 17 patients, no other patients who might have been affected by sarin gas were admitted.

Table 1 describes the demographics of the population, the age mean was 29 years old, most of them were females 65% of the total cases. It is noticeable because of the effects of war that no patient in this sample was obese, but on the contrary, the patients' weights tended to decrease, which may reflect the strict nutritional and poor food supplies under the war circumstances.

**Table 1:** Demographics of the population No correlation was found between outcome and demographics.

Demographics		
Age (years)	Mean	29.1
	Range	4–70
	Under 18	n= 4 (23.5%)
Gender	Male	n= 6 (35.3%)
	Female	n= 11 (64.7%)
Weight (kg)	Mean	52.8
	Range	18–79
Pre-existing disease	Obesity	n= 0 (0%)
Tobacco use	Non-smoker	n= 16 (94.1%)
	Current smoker	n= 1 (5.89%)

Table 2 describes the vital signs of the patients reported upon their presentation, A total of 82.4% of the patients were fully conscious and responsive upon arrival, whereas 64.7% of patients were fully oriented. 94.1% of patients were unstable. 82.4% of patients suffered from tachypnoea and bradycardia. 100% of patients suffered from hypoxia. Except for one case, blood pressure was normal in all patients. All child patients showed blood pressure within normal limits. 64.7% of patients suffered from hyperthermia, while only 11.8% suffered from hypothermia.

**Table 2:** Vital parameters upon patient presentation The vast majority of the patients were unstable, suffering from tachypnea, bradycardia, and hyperthermia.

Vital parameter		
Fully conscious		14 (82.4)
Fully oriented		11 (64.7)
Responsive		14 (82.4)
General condition	Good	1 (5.9)
	Unstable	16 (94.1)
Respiratory rate	Mean	24.8
	Range	22.4–27.1
	Tachypnoea	n= 14 (82.4%)
Oxygen saturation %	Mean	86.12
	Range	72–95
	Hypoxia	17 (100%)
Pulse (bpm)	Mean	54.9
	Range	38–84
	Bradycardia	n= 14 (82.4%)
Blood pressure (mmHg)	Hypotension (adults)	n= 1/13 (7.7%)
	Hypotension (children)	n= 0/4 (0%)
	Range	110/50–130/75
Body temperature (°C)	Mean	38.5°C
	Range	37–39°C
	Normothermia (37.5–38.3 °C)	4 (23.5%)
	Hyperthermia	11 (64.7%)
	Hypothermia	2 (11.8%)

Besides the common respiratory distress symptom, it was noticed that the majority of patients also suffered from intestinal distress, neurological distress, and other signs of sarin intoxication such as nausea, vomiting, abdominal pain, dry mouth, headache, generalized muscle spasms, itchy eyes, constricted pupils, blurred vision, and irregular heart rhythm. All patients except one suffered from constricted pupils. All pupils were fixed (without light reaction). All patients suffered from nystagmus (Table 3).

**Table 3:** Clinical presentation and physical examination of intoxicated patients All of the patients presented with the typical presentation of Sarin gas toxicity in the form of respiratory distress such as dyspnea and difficulty breathing, chest pain, dizziness, epiphora, itchy eyes, nystagmus, and redness of the skin.

Organ system	Symptoms and signs	n (%)
Respiratory	Dyspnoea	17 (100)
	Difficulty breathing	17 (100)
	Intercostal retractions	4 (23.5)
	Rales	0 (0)
	Wheezing	0 (0)
	Roughness	6 (35.3)
Intestinal	Nausea	15 (88.2)
	Vomiting	15 (88.2)
	Oral froth	3 (17.6)
	Abdominal pain	15 (88.2)
	Dry mouth	12 (70.6)
Neurological	Generalized muscle spasms	13 (76.5)
	Headache	15 (88.2)
	Irritation	8 (47.1)
	Dizziness	17(100)
Ophthalmological	Epiphora	17 (100)
	Itchy eyes	14 (82.4)
	Eye pain	17 (100)
	Nystagmus	17 (100)
	Constricted pupils	16 (94.1)
	Dilated pupils	1 (5.9)
	Fixed pupils	17 (100)
	Pupils isokorie	14 (82.4)
	Blurred vision	12 (70.6)
Dermatological	Pruritus	8 (47.1)
	Urticarial rash	9 (52.9)
	Redness	17 (100)
Cardiology examination	Chest pain	17 (100)
	Arrhythmia	16 (94.1)

The main remark of the management of the affected victims was that all patients needed oxygen and atropine, while dexamethasone, bronchodilators, and anti-emetics were needed by the majority of patients. Rantidine (H2 blocker) and analgesics were administered to about half of the patients.

Thirteen patients (76.5%) were discharged from the hospital within 24 hours of admission. Four patients (23.5%) needed to remain at the hospital for more than 24 hours, while the median length of stay was 22.2 hours (with a range of eight to 48 hours) (Table 4).

Eleven patients (64.7%) were discharged in good general condition and with the apparent absence of complications at the time of discharge. However, six patients (35.3%) were transferred to another hospital. Two of them required intubation and mechanical ventilation and were admitted to the ICU in another hospital. One of them was weaned within hours and discharged within 24 hours, while the other was weaned and discharged within 48 hours. The other four patients transferred to another hospital were discharged within 24 hours (Table 4).

**Table 4:** Initial management and outcome of exposed patients Despite the very limited resources, all patients improved, and there were no deaths. Decisions to admit patients to al Rahman or transfer them to another hospital were necessary due to the large number of patients received (55 patients) and the limited space at the hospital (26 beds). Decisions were made based on the intensity of respiratory, gastrointestinal, and neurological symptoms.

Treatment				n (%)
Oxygen				17 (100)
Atropine 0.5 mg/mL				17 (100)
Dexamethasone				14 (82.4)
Bronchodilators				14 (82.4)
Anti-emetics				14 (82.4)
Ranitidine				7 (41.2)
Paracetamol				8 (47.1)
Intubation				2 (11.8)
Outcome				
Hospital admission				17 (100)
ICU admission				2 (11.8)
Length of stay	< 24 h			13 (76.5)
	> 24 h			4 (23.5)
	Mean			22.2 h
	Range			8h to 48h
Improvement				11 (64.7)
Transfer to another hospital				6 (35.3)
Death				0 (0)

## Discussion

This study describes the presentation, treatment, hospital course, and outcome of patients exposed to sarin gas in Khan Shaykhun, Syria. Most symptoms were respiratory, digestive, neurological, cardiological, dermatological, and ophthalmological. This report provides information about the signs and symptoms that can be expected to occur in a large population as a result of acute sarin exposure.

All victims developed significant respiratory disorders indicating severe airway inflammation, as well as digestive symptoms suggesting gastrointestinal inflammation, nervous system disorders, arrhythmia, skin redness, and ocular symptoms suggesting eye inflammation.

Management of patients exposed to sarin gas in austere circumstances included symptomatic treatment using oxygen, atropine, inhaled bronchodilators, anti-emetics, and intravenous corticosteroids. This approach seemed to be effective in managing the symptoms of these patients in an area with poor medical infrastructure and resources, although other medications like pralidoxime (unfortunately not available at the time of exposure) would enhance outcome [11,17].

Although 35.3% of patients in this study were transferred to another hospital, resolution of signs and symptoms was swift in most patients, indicating that even critically ill patients exposed to this toxic gas can most often be discharged within a relatively short period after appropriate supportive care. However, 11.8% of the patients in this sample required mechanical ventilation. Intensive care unit beds may not be available in war-ridden areas with limited resources, challenging the management of critically ill patients and creating the requirement of a well-equipped ambulance transport system staffed with trained professionals.

Many studies have pointed to longer-term damage caused by exposure to sarin gas such as respiratory failure, general loss of bodily function, seizures, brain damage, delayed neurotoxicity, and death [11-15]. This implies that it is important to re-evaluate patients periodically during a longer-term follow-up strategy.

There are several limitations to this study, most of which are the result of complex circumstances. These limitations include lack of resource availability, the overall number of patients, the size of hospital staff, and limited information due to the urgency of patients’ presentations. Important drugs such as pralidoxime and sufficient numbers of ICU beds were not available in the war-ridden region of rural Idlib at the time of the attack and subsequent treatment of patients. The sample size is small because it was practically and ethically impossible due to the conditions of war and the situation of patients to include all victims who presented. The medical staff present at al Rahman Charity Hospital at the time of the overwhelming influx of potential sarin gas attack victims was small compared to what would have been necessary or sufficient to treat all patients on-site. Further, the healthcare workers prioritized treating patients over documenting their clinical states, which explains the relative lack of more robust data.

Survivor bias, because of targeting them with toxic gases, could be present; however, we can defend this possibility as al Rahman Charity Hospital is the closest to the site of the incident, and it is one of several hospitals available in the countryside of Idlib that received victims.

Due to the conditions of war and the huge number of victims of this attack (about 631 [5,6]), we were not able to document more than 2.7% of victim cases or conduct complementary exams such as chest x-rays or electrocardiograms (ECGs). Finally, interrater variability of recognition and interpretation of clinical signs or other variables may have led to sampling bias.

## Conclusions

To the best of our knowledge, this is the first case series report describing the clinical state and management of patients who have been attacked by sarin gas in a war zone. This case series study demonstrates that even in complex humanitarian emergency settings in vulnerable areas with low-resource capacity, the survival rate and prognosis of sarin gas contaminated patients is fair, at least if basic measures are taken and symptomatic treatment is performed with oxygen, atropine, bronchodilators, and corticosteroids. The study provides insight into the clinical presentation, management, and hospital course likely to result from future sarin gas releases. Further investigation and long-term follow-up are required to understand the chronic and/or long-term consequences of such toxic exposure. Further, as physicians, we urge governments to cease the use of chemical weapons immediately.
